# Eating habits and academic performance in secondary school: a cross-sectional analysis in a public school

**DOI:** 10.15649/cuidarte.5050

**Published:** 2025-09-01

**Authors:** Dora Carolina Jaimes Crispín, Silvia Rosa Sigales Ruiz

**Affiliations:** 1 Bacterióloga y Laboratorista Clínico, Especialista en Docencia Universitaria, Magister en Microbiología, Doctoranda en Educación, Bucaramanga, Colombia. E-mail: doracjc@gmail.com Bucaramanga Colombia doracjc@gmail.com; 2 Doctora en Psicopatología, Docente investigadora, Universidad de Colima, México. E-mail: ssigales@ucol.mx Universidad de Colima Colima México ssigales@ucol.mx

**Keywords:** Eating Habits, Academic Performance, Students, Secondary Education, School Health Services, Hábitos Alimentarios, Rendimiento Académico, Estudiantes, Educación Secundaria, Servicios de Salud Escolar, Hábitos Alimentares, Desempenho Acadêmico, Estudantes, Educação Secundária, Saúde Escolar

## Abstract

**Introduction:**

Diet and lifestyle habits have a direct impact on students’ academic performance. Factors such as skipping breakfast, consuming ultra- processed foods, and lack of physical activity can negatively affect school achievement.

**Objective:**

To identify the relationship between eating habits and academic performance among high school students at a public school during 2023.

**Materials and Methods:**

A cross-sectional study was conducted with 189 students. Sociodemographic variables, eating habits, nutritional status (body mass index and hemoglobin levels), sleep duration, and level of physical activity were assessed. Academic performance was measured using grade point averages and Saber test scores. Descriptive and multivariate statistical analyses were performed.

**Results:**

A total of 60.85% of students reported never eating breakfast or doing so less than once a week, with this behavior being more prevalent among those with lower academic performance. Sleeping eight hours per night (aOR = 1.43; 95% CI: 0.62–3.28) and engaging in regular physical activity (aOR = 5.81; 95% CI: 1.05–31.95) were associated with better academic results. No significant differences were found in terms of body mass index, hemoglobin levels, or consumption of vegetables, fruits, cereal grains, proteins, snacks, and sugar-sweetened beverages.

**Discussion:**

Various studies have demonstrated that nutrition plays a key role in cognitive development.

**Conclusion:**

Healthy lifestyle habits such as regular breakfast consumption, adequate sleep, and physical activity positively influence academic performance. Therefore, it is essential to strengthen school-based strategies that promote a balanced diet and healthy lifestyle to enhance student achievement.

## Resumen

**Introducción:**

La alimentación y los hábitos de vida tienen un impacto directo en el rendimiento académico de los estudiantes. Factores como la omisión del desayuno, el consumo de alimentos ultra procesados y la falta de actividad física pueden afectar el desempeño escolar.

**Objetivo:**

Identificar la relación entre los hábitos de consumo de alimentos y el rendimiento académico en estudiantes de secundaria de un colegio público durante 2023.

**Materiales y métodos:**

Estudio transversal con 189 estudiantes. Se evaluaron variables sociodemográficas, hábitos alimenticios, estado nutricional (índice de masa corporal y nivel de hemoglobina), horas de sueño y nivel de actividad física. El rendimiento académico se midió mediante el promedio de calificaciones y las pruebas Saber. Se realizaron análisis estadísticos descriptivos y multivariados.

**Resultados:**

Se identificó que el 60,85% de los estudiantes nunca desayuna o lo hace menos de una vez a la semana, con una mayor prevalencia en aquellos con menor rendimiento académico. Dormir ocho horas (ORa=1,43; IC95%: 0,62-3,28) y realizar actividad física regularmente se asoció con mejores calificaciones (ORa=5,81; IC95%: 1,05-31,95). No se encontraron diferencias significativas en el IMC, la hemoglobina, el consumo de verduras, frutas, cereales, proteínas, ni de bocadillos y bebidas azucaradas.

**Discusión:**

Diferentes estudios han identificado que la nutrición es relevante para el desarrollo cognitivo.

**Conclusiones:**

Los hábitos de vida saludables, como desayunar regularmente, dormir adecuadamente y realizar actividad física influyen en el rendimiento académico, por lo que es necesario fortalecer estrategias escolares que promuevan una alimentación equilibrada y un estilo de vida saludable para mejorar el desempeño estudiantil.

## Resumo

**Introdução:**

Hábitos alimentares e de estilo de vida impactam diretamente o desempenho acadêmico dos alunos. Fatores como pular o café da manhã, consumir alimentos ultraprocessados e a falta de atividade física podem afetar o desempenho acadêmico.

**Objetivo:**

Identificar a relação entre hábitos alimentares e desempenho acadêmico em alunos do Ensino Médio de uma escola pública durante o ano de 2023.

**Materiais e métodos:**

Estudo transversal com 189 alunos. Foram avaliados variáveis sociodemográficas, hábitos alimentares, estado nutricional (índice de massa corporal e nível de hemoglobina), horas de sono e nível de atividade física. O desempenho acadêmico foi mensurado por meio da média ponderada de notas e dos testes Saber. Análises estatísticas descritivas e multivariadas foram realizadas.

**Resultados:**

Identificou- se que 60,85% dos alunos nunca tomam café da manhã ou o fazem menos de uma vez por semana, com maior prevalência entre aqueles com menor desempenho acadêmico. Dormir oito horas (ORa=1,43; IC 95%: 0,62-3,28) e praticar atividade física regular foram associados a melhores notas (ORa=5,81; IC 95%: 1,05-31,95). Não foram encontradas diferenças significativas no índice de massa corporal, hemoglobina, consumo de vegetais, frutas, cereais, proteínas ou lanches e bebidas açucaradas.

**Discussão:**

Diferentes estudos identificaram que a nutrição é relevante para o desenvolvimento cognitivo.

**Conclusões:**

Hábitos de vida saudáveis, como tomar café da manhã regularmente, dormir adequadamente e praticar atividade física influenciam o desempenho acadêmico, por isso é necessário fortalecer estratégias escolares que promovam uma dieta balanceada e um estilo de vida saudável para melhorar o desempenho dos alunos.

## Introduction

Nutrition is a key factorin the physical and cognitive development of students, as it directly affects their concentration, memory, and academic performance. According to the World Health Organization (WHO), a balanced diet can improve academic performance by up to 20%, whereas a poor diet can lead to fatigue, lack of attention, and reduced learning capacity^1^. Despite growing evidence on the relationship between nutrition and academic performance, many educational settings, especially public schools, face challenges related to students' access to healthy foods and proper eating habits^2^,^3^. 

Recent studies have shown that students who regularly eat breakfast perform 30% better on math and reading tests compared to those who skip this meal^4^. Furthermore, a 2022 report by the Food and Agriculture Organization of the United Nations (FAO) indicates that approximately 40% of adolescents in Latin America do not follow a balanced diet, which negatively impacts their academic performance^5^. In socioeconomically vulnerable contexts, limited access to nutritious food increases the consumption of ultra-processed products high in sugars and fats, thereby compromising students' cognitive development and academic performance^6^,^7^. 

In public schools, the quality of food available at home and through school feeding programs can determine students’ nutritional intake. Research has shown that diets rich in protein, essential fatty acids, and micronutrients such as iron and zinc enhance learning capacity, while excessive consumption of sugars and refined carbohydrates is associated with decreased academic performance^4^,^6^,^8^. However, in many countries, the food provided in schools remains deficient^3^, highlighting the need to assess how eating habits affect education in these contexts. 

This study aims to identify the relationship between food consumption habits and academic performance among high school students in a public school. To this end, we will analyze students' dietary patterns and their impact on their school achievement, with the goal of generating evidence that can contribute to the implementation of strategies to improve student nutrition and optimize academic outcomes. 

## Materials and Methods

**Study design**

An observational, quantitative, cross-sectional, and analytical study was conducted with the participation of students from a public educational institution during 2023.

**Population and sample**

The study involved ninth-grade students from an educational institution. Out of a total of 368 students, 189 participated in the study. The sample size was calculated assuming an alpha error of 0.05 and a beta of 0.80, based on a study that aimed to identify factors associated with academic performance. That study reported that 76% of participants had an average level of academic performance, 20% had healthy eating habits, and 76% required changes in their diet^9^. The sample size calculation was performed in EPIDAT 3.1 software. A convenience sampling method was employed, with students participating voluntarily. 

**Instruments**

The Promoting Eating at School^10^ questionnaire was administered to explore food consumption patterns, perceived healthiness of snacks, activities during school recess, eating and purchasing behaviors, and weekly food expenditure outside the school setting. recess, eating and purchasing behaviors, and weekly food expenditure outside the school setting. The Healthy Eating Index for the Spanish Population (IASE)- Consumption Frequency Questionnaire (CFQ)^9^,^11^ was used to assess the frequency of consumption of various food groups. Additionally, the Harvard Healthy Eating Plate^12^ model was used to determine the proportion of vegetables, fruits, cereal grains, and healthy proteins in students' lunches. 

Cognitive processes were assessed using tests previously applied in various studies in this population and the country, including the Trail Making Test Part A (TMT Part A) for attention^13^, TMT Part B, Logical Thinking Test (LTT)^14^, Auditory Memory Test (AMT)^15^, and Modified Wisconsin Card Sorting Test (M-WCST)^16^. 

Nutritional status was assessed through height measurement using a wall-mounted stadiometer and weight measurement with a calibrated digital scale, allowing for body mass index (BMI) calculation. Hemoglobin levels were measured with a hematology analyzer as part of a complete blood count. 

Academic performance was measured using the Grade Point Average (GPA) of the subjects each student was taking at the institution. The Saber 9 standardized test assessed competencies in Language, Mathematics, Natural Sciences, and Citizenship^17^. 

**Variables**

Sociodemographic characteristics (sex, age, socioeconomic status, housing, parents' educational level, and parents' income). Habits (sleep duration, screen time on information technology and telecommunication devices, food consumption habits during the school day [breakfast, lunch, snacks, sugary drinks, bites]). Anthropometric measurements (weight, height, and BMI). Hemoglobin levels. Food portion consumption (intake of vegetables, fruits, cereal grains, proteins). Cognitive assessment tests (academic GPA, TMT Part A, TMT Part B, LTT, AMT, and M-WCST). 

**Data analysis**

A descriptive analysis was conducted for the variables of interest. Categorical characteristics were described using percentages and absolute frequencies. For numerical variables, normality was assessed using the Shapiro–Francia test, and results are presented as medians and interquartile ranges (IQR). Combined bar charts were generated using Microsoft Excel. 

A comparison was made by grade point averages (GPAs); students were grouped into terciles based on their GPAs. Terciles 1 and 2 were combined and compared with tercile 3. Pearson’s Chi-square test and Fisher’s exact test were used to compare categorical variables, while the Mann-Whitney U test was applied to numerical variables. Crude odds ratios (cOR) were calculated using logistic regression, with corresponding 95% confidence intervals (95% CI). A multivariate logistic regression model was developed following the steps proposed by Hosmer and Lemeshow. Model fit was assessed. Data analysis was performed using Microsoft Excel and Stata version 17. The dataset is available on Mendeley Data^18^. 

**Ethical considerations**

In accordance with Resolution 8430 of 1993^19^, the study was classified as risk-free, as it involved the use of surveys and tests. Ethical principles were upheld^20^, and assent and informed consent were obtained, ensuring respect for participants' autonomy. The research was approved by the Bioethics Committee of Universidad Iberoamericana de México, UNINI, under Minute No. 001, dated November 2, 2022. 

## Results

**Students' characteristics**

Of the total of 189 participants, 43.92% (83) were female and 56.08% (106) male, 75.13% (142) were between 14 and 16 years old, 61.90% (117) belonged to socioeconomic stratum 1, and 40.74% (77) lived in rented housing. A comparison was made between the participants with GPAs in the lower and middle terciles, ranging from 43.21 to 77.42 (124), and those in the upper tercile, who had higher grades, ranging from 77.57 to 94.5 (62). When comparing sociodemographic variables, no statistically significant differences were observed, except for the parents' level of education; students with better GPAs reported that their parents had either “None” education or “Elementary” education. 

In terms of students’ habits, significant differences were observed in sleep duration: students who reported sleeping exactly 8 hours had a higher likelihood of being in the group with better academic performance, while those who slept seven hours or less or nine hours or more had a lower tendency to be in this group. In addition, students who reported engaging in physical activity were 4.76 times more likely (95% CI: 1.06–21.33) to belong to the group with the highest grades. 

Regarding nutritional status based on BMI, 67.72% (128) of participants had a normal weight, 17.99% (34) were underweight, 11.11% (21) were overweight, and 3.17% (6) were classified as obese. No statistically significant differences were found between GPA groups regarding nutritional status (p = 0.410) with hemoglobin levels ranging from 10.5 to 11.9g/dL (55.56% [105]) and equal to or greater than 12 g/dL (44.44% [84]) (see Table 1). 

**Table 1:** Student characteristics and habits

Variable	Total (189)	Grade point average		p-value	cOR (95% CI)
		Terciles 1-2 43.21-77.42 (124)	Tercile 3 77.57-94.5 (62)		
Sex				0.217	
Female	43.92(83)	45.97(57)	38.71(24)		1
Male	56.08(106)	54.03(67)	61.29(38)		1.34 (0.72 - 2.50)
Age				0.580	
10-13	1.06(2)	1.61(2)	0		1
14-16	75.13(142)	75.81(94)	72.58(45)		0.93 (0.08 - 10.60)
17-21	23.81(45)	22.58(28)	27.42(17)		1.21 (0.10 - 14.42)
Socioeconomic stratum				0.775	
1	61.90(117)	63.71(79)	59.68(37)		1
2	30.16(57)	28.23(35)	33.87(21)		1.28 (0.65 - 2.49)
3/5	7.94(15)	8.06(10)	6.45(4)		0.85 (0.25 - 2.90)
Housing				0.064	
Owner	30.69(58)	34.68(43)	22.58(14)		1
Tenant	40.74(77)	41.94(52)	38.71(24)		1.41 (0.65 - 3.07)
Shared housing	28.57(54)	23.39(29)	38.71(24)		2.54 (1.13 - 5.71)
Parents' educational level				0.029	
None	20.63(39)	16.94(21)	29.03(18)		0
Elementary	46.03(87)	43.55(54)	51.61(32)		0.69 (0.32 - 1.48)
Secondary	26.98(51)	32.26(40)	14.52(9)		0.26 (0.10 - 0.68)
University	6.35(12)	7.26(9)	4.84(3)		0.38 (0.09 - 1.65)
Parents' income				0.432	
Low	60.32(114)	58.87(73)	64.52(40)		1
Middle	37.57(71)	37.90(47)	35.48(22)		0.85 (0.45 - 1.61)
High	2.12(4)	3.23(4)	0		1
Sleep hours				0.037	
≤ 7 hours	28.04(53)	31.45(39)	22.58(14)		1
8 hours	62.43(118)	56.45(70)	74.19(46)		1.83 (0.89 - 3.74)
≥ 9 hours	9.52(18)	12.10(15)	3.23(2)		0.37 (0.07 - 1.83)
Screen time				0.634	
< 2 hours	26.46(50)	24.19(30)	30.65(19)		1
2 hours	31.75(60)	30.65(38)	33.87(21)		0.87 (0.39 - 1.91)
3 hours	17.46(33)	18.55(23)	14.52(9)		0.61 (0.23 - 1.61)
> 3 hours	24.34(46)	26.61(33)	20.97(13)		0.62 (0.26 - 1.47)
Physical activity				0.037	
No	10.22(19)	13.71(17)	3.23(2)		1
Yes	89.95(170)	86.29(107)	96.77(60)		4.76 (1.06 - 21.33)
Physical activity (hours)				0.074	
< 1 hours	11.70(20)	11.21(12)	13.11(8)		1
1 hours	32.16(55)	38.32(41)	21.31(13)		0.47 (0.15 - 1.41)
> 1 hours	56.14(96)	50.47(54)	65.57(40)		1.11 (0.41 - 2.97)
BMI				0.410	
Underweight	17.99(34)	17.74(22)	19.35(12)		1
Normal	67.72(128)	66.94(83)	69.35(43)		0.94 (0.42 - 2.10)
Overweight	11.11(21)	10.48(13)	11.29(7)		0.98 (0.31 - 3.14)
Obesity	3.17(6)	4.84(6)	0		1
Hemoglobin g/dL				0.347	
10.5-11.9	55.56(105)	58.87(73)	51.61(32)		1
≥12	44.44(84)	41.13(51)	48.39(30)		1.34 (0.72 - 2.47)

cOR: Crude Odds Ratio. BMI: Body Mass Index. g/dL: grams per deciliter. p-value: Pearson's Chi-square test or Fisher's Exact test.

**Consumption habits**

 The majority of participants (60.85% [115]) reported "never eating breakfast or doing so less than once per week." In the group with the highest GPAs, 75.81% (47) reported this behavior, compared to 54.03% (67) in the group with the lowest GPAs. It is worth noting that, according to the survey, these students wake up very early, often needing to commute in order to arrive at school by 6:00 a.m., and most reported not having breakfast. Only 22.75% (43) reported eating breakfast every day of the week. Regarding lunch habits, 47.60% (90) reported eating lunch seven days a week, another 47.62% (90) reported doing so between one and six days per week, and 4.76% (9) stated they never ate lunch or ate lunch less than a day per week (see Table 2).

 The consumption of snacks and sugary drinks three or more times per day was more frequent among students in the higher GPA group; however, this association was not statistically significant (p > 0.05). As for the perception of snack foods, 79.89% (151) of participants considered them to be unhealthy. In terms of food procurement, 50.79% (96) reported a combination of sources, including purchasing food from the school cafeteria, bringing food from home, and buying food elsewhere. Additionally, 36.51% (69) reported purchasing food from the school cafeteria four or more times per week.

**Table 2:** Consumption habits in the school environment

Variable	Total (189)	Grade point average		p-value	cOR (95% CI)
		Terciles 1-2 43.21-77.42 (124)	Tercile 3 77.57-94.5 (62)		
Breakfast per week				0.016	
Never or < 1 day	60.85(115)	54.03(67)	75.81(47)		1
1-6 days	16.40(31)	20.16(25)	9.68(6)		0.34 (0.13 - 0.89)
7 days	22.75(43)	25.81(32)	14.52(9)		0.40 (0.17 - 0.91)
Lunch per week				0.511	
Never or < 1 day	4.76(9)	3.23(4)	6.45(4)		1
1-6 days	47.62(90)	47.58(59)	50.00(31)		0.52 (0.12 - 2.24)
7 days	47.62(90)	49.19(61)	43.55(27)		0.44 (0.10 - 1.90)
Snack consumption				0.079	
Never or < 1 day	8.99(17)	12.10(15)	3.23(2)		1
1 per day	1058(20)	11.29(14)	8.06(5)		2.67 (0.44 - 16.11)
2 per day	33.33(63)	35.48(44)	30.65(19)		3.23 (0.67 - 15.57)
3 or more per day	47.09(89)	41.13(51)	58.06(36)		5.29 (1.13 - 24.59)
Sugary drink consumption				0.074	
Never or < 1 day	6.35(12)	7.26(9)	4.84(3)		1
1 per day	13.23(25)	16.13(20)	6.45(4)		0.60 (0.11 - 3.25)
2 per day	27.51(52)	29.84(37)	22.58(14)		1.13 (0.26 - 4.81)
3 or more per day	52.91(100)	46.77(58)	66.13(41)		2.12 (0.54 - 8.31)
Perception of snack foods				0.096	
Unhealthy	79.89(151)	76.61(95)	85.48(53)		1
Healthy	16.93(32)	20.97(26)	9.68(6)		0.41 (0.16 - 1.06)
Very healthy	3.17(6)	2.42(3)	4.84(3)		1.79 (0.35 - 9.19)
Source of food for in-school consumption				0.477	
Brought from home	9.52(18)	10.48(13)	6.45(4)		0.55 (0.16 - 1.82)
Bought at the school cafeteria	30.16(57)	32.26(40)	25.81(16)		0.71 (0.35 - 1.46)
Bought elsewhere	9.52(18)	8.06(10)	12.90(8)		1.43 (0.51 - 3.98)
Combination of the above sources	50.79(96)	49.19(61)	54.84(34)		1
Frequency of purchasing food at the school cafeteria				0.555	
Never	3.70(7)	2.23(4)	4.84(3)		1
1-2 times per week	25.93(/49)	24.19(30)	29.03(18)		0.80 (0.16 - 3.99)
3 times per week	33.86(64)	37.10(46)	27.42(17)		0.49 (0.09 - 2.43)
4 or more times per week	36.51(69)	35.48(44)	38.71(24)		0.72 (0.15 - 3.52)

cOR: Crude Odds Ratio. BMI: Body Mass Index. P-value: Pearson's Chi-square test or Fisher's Exact test.

Regarding vegetable consumption, 20.95% (39) of the students reported that vegetables accounted for less than 5% of their plate, while 63.44% (118) reported a proportion between 5% and 10%. The median percentage of vegetable intake in both GPA groups was 5%. The measure of association indicated that participants were 5% less likely to be in the higher GPA group (cOR = 0.95 [0.92-0.99]) (See Table 3). 

With respect to fruit consumption, 17.74% (33) of students reported that fruits made up less than 5% of their plate, while 67.20% (125) reported a proportion between 5% and 10%. Only 6.45% (12) indicated that fruits represented more than 50% of their plate. Cereal grain consumption accounted for less than 5% of the plate in 71.51% (133) of cases. Among students with higher GPAs, this was reported by 80.65% (50), compared to 66.94% (82) among those with lower GPAs. Protein intake between 5% and 10% of the plate was reported by 60.75% (113) of students overall, 64.52% (40) in the high GPA group, and 58.87% (73) in the low GPA group. 

**Table 3:** Percentage of food types on the plate

Variable	Total (189)	Grade point average		p-value	cOR (95% CI)
		Terciles 1-2 43.21-77.42 (124)	Tercile 3 77.57-94.5 (62)		
Vegetables					
Median (IQR)	5(5- 10)	5(5- 10)	5(0- 5)	0.0266	0.95 (0.92 -0.99)
Ranges				0.050	
< 5	20.97(39)	18.55(23)	25.81(16)		1
5-10	63.44(118)	61.29(76)	67.74(42)		0.79 (0.37 - 1.66)
11-50	10.75(20)	12.90(16)	6.45(4)		0.35 (0.10 - 1.27)
> 50	4.84(9)	7.26(9)	0		1
Fruits					
Median (IQR)	5(5- 10)	5(5- 10)	5(5- 5)	0.0618	0.97 (0.94 - 1.00)
Ranges				0.097	
< 5	17.74(33)	18.55(23)	16.13(10)		1
5-10	67.20(125)	62.10(77)	77.42(48)		1.43 (062 - 3.27)
11-50	8.60(16)	10.48(13)	4.84(3)		0.53 (0.12 - 2.28)
> 50	6.45(12)	8.87(11)	1.61(1)		0.20 (0.02 - 1.84)
Cereal grains					
Median (IQR)	0(0- 5)	0(0- 5)	0(0- 0)	0.0269	0.96 (0.93 - 0.99)
Ranges				0.060	
< 5	71.51(133)	66.94(83)	80.65(50)		1
5-10	14.52(27)	14.52(18)	14.52(9)		0.83 (0.34 - 1.98)
11-50	10.22(19)	12.90(16)	4.84(3)		0.31 (0.08 - 1.21)
> 50	3.76(7)	5.65(7)	0		1
Protein					
Median (IQR)	5(5- 5)	5(5- 12.5)	5(5- 10)	0.0751	0.97 (0.95 - 0.99)
Ranges				0.100	
< 5	18.82(35)	16.13(20)	24.19(15)		1
5-10	60.75(113)	58.87(73)	64.52(40)		0.73 (0.33 - 1.58)
11-50	13.98(26)	16.13(20)	9.68(6)		0.40 (0.12 - 1.24)
> 50	6.45(12)	8.87(11)	1.61(1)		0.12 (0.01 - 1.04)

cOR: Crude Odds Ratio. p-value (categorical variables- Quantitative variables): Pearson's Chi-square test or Fisher's Exact test. P-value (numerical variables): Mann-Whitney U test.

**Figure 1:**
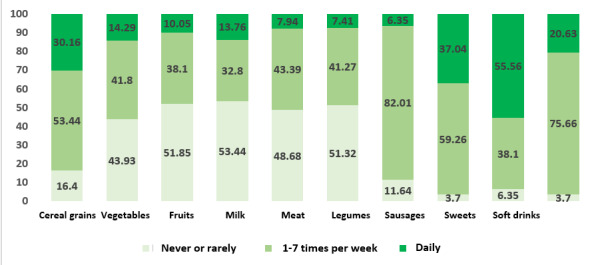
Prevalence of food consumption among students

According to reported consumption frequency, students indicated daily intake of the following food items: soft drinks (55.56% [103]), sweets (37.04% [70]), cereal grains (30.16% [56]), fats (20.63% [39]), vegetables (14.29% [26]), milk (13.76% [26]), fruits (10.05% [18]), meat (7.94% [15]), legumes (7.41% [14]), and processed meats/sausages (6.35% [12]). Among those who reported never or rarely consuming vegetables, fruits, milk, meat, and legumes, the percentages ranged between 43.92% and 53.44% (see Figure 1). 

**Cognitive assessment tests**

The cognitive competence assessment yielded the following median results: 62 seconds (IQR: 37- 103) for the TMT Part A, and 105 seconds (IQR: 72-144) for the TMT Part B. For the Logical Thinking Test (LTT), yielded a median of 4 points (IQR: 3-5), for the Auditory Memory Test (AMT) a median of 56 points (IQR: 47-64), and for the M-WCST a median of 17 points (IQR: 19-33). In the Saber test, students achieved a median score of 34 (IQR: 30–40). When stratified by academic performance, the group with higher GPAs had a median of 38 (IQR: 32-45), compared to a median of 33 (IQR: 30-39) in the lower-GPA group, with a statistically significant difference (p = 0.0004). The measure of association indicated that for each one-point increase in the Saber test score, students had a 9% higher likelihood of belonging to the group with better GPAs (cOR = 1.09; 95% CI: 1.04–1.14). (See Table 4). 

**Table 4:** Comparison of cognitive assessment tests, Saber test, and GPA, Median (Interquartile Range, IQR)

Variable	Total (189)	Grade point average		p-value	cOR (95% CI)
		Terciles 1-2 43.21-77.42 (124)	Tercile 3 77.57-94.5 (62)		
TMT Part A	62 (37-103)	63 (36-103)	59.5 (41-105)	0.7937	1.00 (0.99 - 1.00)
TMT Part B	105 (72-144)	102 (70.5-141.5)	115 (72-163)	0.3112	1.00 (0.99 - 1.00)
LTT	4 (3-5)	4 (3-5)	4 (3-5)	0.5202	1.04 (0.88 - 1.23)
AMT	56 (47-64)	58 (48-64.5)	55 (45-63)	0.1851	0.98 (0.96 - 1.00)
M-WCST	17 (19-33)	27 (18.5-32)	28 (19-34)	0.4066	1.01 (0.98 - 1.05)
Saber Test	34 (30-40)	33 (30-39)	38 (32-45)	**0.0004**	**1.09 (1.04 - 1.14)**

TMT Part A: Trail Making Test Part A (attention); TMT Part B: Trail Making Test Part B; Logical Thinking Test (LTT); Auditory Memory Test (AMT); M-WCST: Modified Wisconsin Card Sorting Test. P-value: Mann-Whitney U test.

**Multivariate analysis**

Two factors were found to be significantly associated with a higher likelihood of belonging to the upper tercile (i.e., students with the highest GPAs). The first was engagement in physical activity, with an adjusted odds ratio (aOR) of 5.81 (95% CI: 1.05–31.95; p = 0.043). The second was performance on the Saber test, with an aOR of 1.10 (95% CI: 1.04–1.15; p < 0.001). Although the consumption of vegetables, fruits, cereal grains, and proteins did not show a statistically significant difference, a potential positive trend was observed between higher protein intake and the likelihood of being in the upper GPA tercile. (See Table 5).

**Table 5:** Multivariate logistic regression analysis of factors associated with belonging to the upper GPA tercile

Variable	aOR (95% CI)	p-value
% Vegetable	0.95 (0.89 - 1.02)	0.198
% Fruits	1.00 (0.95 - 1.05)	0.836
% Cereal grains	0.98 (0.94 - 1.03)	0.585
% Proteins	1.02 (0.97 - 1.06)	0.374
Physical activity		
No	1	
Yes	5.81 (1.05 - 31.95)	**0.043**
Parents' level of education		
None	1	
Primary	0.61 (0.25 - 1.49)	0.285
Secondary	0.38 (0.11 - 1.24)	0.110
Professional	0.46 (0.08 - 2.67)	0.389
Sleep hours		
≤ 7 hours	1	
8 hours	1.43 (0.62 - 3.28)	0.394
≥ 9 hours	0.26 (0.03 - 1.71)	0.162
Saber Test Score	1.10 (1.04 - 1.15)	**<0.001**

aOR: Adjusted Odds Ratio. 95% CI: 95% Confidence Interval. P-value: Wald test. %: percentage.

## Discussion

The results of this study did not reveal a significant relationship between certain food consumption habits and academic performance among high school students in a public school. However, it was found that students who reported engaging in regular physical activity were more likely to achieve higher grades. These findings are consistent with previous research that underscores the role of physical activity in supporting learning and cognitive functioning^21^-^22^, particularly emphasizing the benefits of engaging in 30 to 90 minutes of physical activity per day. Such activity has also been linked to convenient time management and study planning. 

In terms of eating habits, it was observed that the majority of students did not follow healthy consumption patterns. Specifically, 60.85% reported never eating breakfast or doing so less than once per week. This pattern was more prevalent among students with lower GPAs, suggesting that skipping breakfast may be associated with reduced academic performance. Prior studies have demonstrated that breakfast is essential for enhancing concentration and cognitive performance, as it provides glucose, a key energy source for brain function^4^,^8^,^23^,^24^. Additionally, a study conducted in Korea^25^ reported that breakfast eating (OR= 2.34; 95%CI: 2.20-2.48), fruit intake (OR= 1.73; 95%CI: 1.62-1.86), vegetable intake (OR= 1.48; 95%CI: 1.37-1.61), and milk consumption (OR= 1.35; 95%CI: 1.28-1.43) were positively associated with higher school grades. In contrast, the consumption of fast food (OR= 0.83; 95%CI: 0.72-0.96), soft drinks (OR= 0.42; 95%CI: 0.38- 0.46), instant noodles (OR= 0.62; 95%CI: 0.55-0.70), and confectioneries (OR= 0.86; 95%CI: 0.80- 0.93) was associated with lower grades. Similarly, a study conducted in Chile^26^ found that the consumption of unhealthy snacks was associated with a lower likelihood of passing language (OR= 0.44; 95% CI: 0.23-0.85), and mathematics (OR= 0.34; 95%CI: 0.19-0.64) courses. 

However, no statistically significant differences were found between the consumption of snacks and sugary drinks and GPA, a result that contrasts with findings from a recent study conducted in Spain. That study reported that students with high consumption levels of ultra-processed foods exhibited poor academic performance in language, mathematics, and English subjects^27^. In contrast, a study conducted in Brazil identified a decrease in the consumption of ultra-processed foods following the COVID-19 pandemic; however, there is a need to continue improving purchasing practices by promoting adherence to nutritional guidelines^28^. 

In the present study, it is noteworthy that a high percentage of students perceived ultra-processed foods as unhealthy. This result suggests the need to improve the availability of food offered in the school environment. Additionally, the unexpected relationship between parents' educational level and students' academic performance suggests that other factors, such as family support and personal motivation, may play a more influential role in educational outcomes.

Body mass index (BMI) analysis showed that 67.72% of the students had a normal nutritional status, while the remaining participants were classified as underweight (17.99%), overweight (11.11%), or obese (3.17%). However, no significant differences were observed between BMI and academic performance. This finding suggests that nutritional status alone is not a determinant predictor of school performance, and that other factors such as diet quality and study habits may be more relevant in this population. Notably, a study conducted in Peru found that video game disorder was associated with low levels of physical activity and poor eating habits^29^. It would therefore be useful to explore whether a similar relationship exists in the population of the present study, as a potential line for future research. 

Food consumption patterns within the school environment reflected a concerning trend: 79.89% of students perceived the available snacks as unhealthy, and 55.56% reported consuming sugary drinks daily. Although snack and sugary beverage consumption showed a slight trend toward association with better GPA, this relationship was not statistically significant. A study conducted in Chile reported that schoolchildren showed a low frequency of healthy food consumption; in addition, low dairy intake and skipping breakfast were associated with poorer performance in language and mathematics^30^. 

These results suggest the need for further analysis of the effects of processed food consumption on learning and the importance of exploring strategies to improve the nutritional quality of food available in educational institutions. 

Another relevant finding was the association between parents' educational level and students' academic performance. Students whose parents had only primary education or no formal education showed a higher proportion of high grades compared to those whose parents had attained higher educational levels. This counterintuitive result may be explained by factors such as family support or greater personal effort by students in contexts with limited access to educational resources. Previous research has shown that family environment and educational support strategies can partially offset socioeconomic disparities in academic achievement^31^. 

Although the percentage of vegetables, fruit, cereal grains, and protein consumption did not show statistically significant associations with academic performance, a trend was observed suggesting that higher protein intake may be linked to better academic performance. This finding is consistent with research that highlights the role of essential amino acids in brain function and learning processes^6^. For future studies, it is suggested to characterize the amount of physical activity, as well as to compare findings across public and private school settings, such as the study by Arias et al., where it was identified students attending public schools were more likely to be physically inactive (adjusted Prevalence Ratio [PR] = 1.40; 95% CI: 1.03–1.92)^28^. It would also be good to investigate the effect on academic performance of other variables such as breastfeeding history^32^-^33^, maternal caloric intake during pregnancy^34^, domestic violence^35^, and lifestyle changes through structured interventions^36^–^38^. 

Among the study's strengths are the use of validated instruments and the application of multivariate analysis. Among the limitations, the use of convenience sampling should be taken into account, which may introduce selection bias, as those who chose to participate may have particular characteristics, such as a higher consumption of snacks or sugary drinks. For future studies, census sampling or simple random sampling is recommended, where feasible. Comparisons by socioeconomic stratum can also be considered, as this factor has been shown to be relevant in other studies^39^. 

## Conclusions

 Physical activity and Saber test scores were the most relevant factors associated with belonging to the upper GPA tercile. Although no statistically significant associations were found between the consumption of specific food groups and academic performance, a trend was observed suggesting a potential positive impact of protein intake on learning outcomes.

 In this sense, the results of this study highlight the need to implement strategies that promote healthy habits among students, such as encouraging the regular consumption of nutritious breakfasts, reducing the intake of ultra-processed foods, and fostering the practice of physical activity. Such measures may not only contribute to the overall well-being of students but also enhance their academic performance and educational opportunities.
